# Effects of Dietary Potassium Diformate Supplementation on Growth Performance, Nutrient Digestibility, Gastrointestinal pH, Jejunal Morphology, Digestive Enzyme Activity, and Antioxidant Status in Weaned Piglets

**DOI:** 10.3390/ani15192916

**Published:** 2025-10-07

**Authors:** Lina Zhang, Yong Cheng, Li Lu, Songtao Zhong, Tiande Zou, Mingren Qu, Jun Chen

**Affiliations:** Jiangxi Province Key Laboratory of Animal Nutrition and Feed, Jiangxi Province Key Innovation Center of Integration in Production and Education for High-Quality and Safe Livestock and Poultry, Jiangxi Agricultural University, Nanchang 330045, China

**Keywords:** antioxidant status, digestive enzyme activity, gastrointestinal pH, growth performance, jejunal morphology, nutrient digestibility, weaned piglets, potassium diformate

## Abstract

**Simple Summary:**

In modern swine production, the nursery phase is a critical stage during which weaned pigs exhibit underdeveloped gastrointestinal tracts. Organic acids have potential benefits in supporting the development of weaned piglets and aiding them through this challenging period. This study demonstrated that dietary supplementation with 1.8% potassium diformate reduced feed conversion ratio of weaned piglets, which was linked to enhanced nutrient digestibility, reduced pH in the anterior gastrointestinal tract, and improved jejunal morphology.

**Abstract:**

This study evaluated the effects of dietary potassium diformate supplementation on growth performance, nutrient digestibility, gastrointestinal pH, jejunal morphology, digestive enzyme activity, and antioxidant status of weaned piglets in a 28-day trial. Twenty-four weaned piglets were selected and, after a 4-day adaptation period, randomly assigned to 4 treatment groups (n = 6). The dietary treatments included a control diet (basal diet) and 3 diets supplemented with 0.6%, 1.2%, or 1.8% potassium diformate in the basal diet. The results indicated that the feed conversion ratio (FCR) of piglets was reduced by all three potassium diformate supplementation levels compared to the control group (*p* < 0.05). Additionally, the FCR was decreased in piglets fed the 1.8% potassium diformate-supplemented diet compared to those fed the 1.2% potassium diformate-supplemented diet (*p* < 0.05). Piglets fed the three potassium diformate-supplemented diets exhibited higher apparent total tract digestibility (ATTD) of dry matter and crude protein than the control group (*p* < 0.05). The 1.8% potassium diformate groups also showed increased ATTD of calcium and phosphorus compared to the control group (*p* < 0.05). Supplementation with 1.2% or 1.8% potassium diformate reduced the digesta pH in the proximal stomach, distal stomach, and duodenum, while increased jejunal villus height (VH), VH/crypt depth (VH/CD) ratio, and catalase and total superoxide dismutase activities in the jejunal mucosa compared to the control group (*p* < 0.05). The 1.2% potassium diformate group showed higher α-amylase activity than the control group (*p* < 0.05). Correlation analysis revealed that FCR negatively correlated with ATTD of dry matter, crude protein, calcium, phosphorus, and jejunal VH, while positively correlating with digesta pH in the proximal stomach (*p* < 0.05). The ATTD of dry matter negatively correlated with digesta pH in the proximal stomach, distal stomach, and duodenum, and positively correlated with jejunal VH/CD ratio and catalase activity (*p* < 0.05). The ATTD of crude protein negatively correlated with digesta pH in the proximal stomach, distal stomach, and duodenum (*p* < 0.05). Collectively, dietary supplementation with 1.8% potassium diformate reduced FCR of weaned piglets, which was associated with enhanced nutrient digestibility, reduced pH in the anterior gastrointestinal tract, and improved jejunal morphology.

## 1. Introduction

Globally, piglet mortality and morbidity present significant welfare challenges and contribute substantially to economic losses [1,2,3]. Gastrointestinal disorders are among the leading causes of morbidity, mortality, and reduced productivity in piglets [4]. In modern swine production, the nursery phase is a critical stage during which weaned pigs exhibit underdeveloped gastrointestinal tracts [5,6]. Insufficient secretion of gastric hydrochloric acid is a key factor contributing to the challenges faced by weaned piglets [4]. Additionally, weaned piglets experience inadequate digestive enzyme activity [7], underdeveloped antioxidant system [8], and immature intestinal villus development [9]. These factors collectively compromise the growth performance of weaned piglets.

Potassium diformate is an organic acid consisting of formic acid and formate, linked through hydrogen bonding and covalent interactions [10]. The mode of action of potassium diformate can be primarily attributed to its ability to lower gastrointestinal pH and its antimicrobial properties, which originate from its simple and unique molecular structure (HCOOH·HCOOK) [11,12,13]. Potassium diformate has been reported as a feed additive for weaned piglets [14,15]. Zhou et al. (2015) found that dietary supplementation with 1% potassium diformate for 35 days improved growth performance and nutrient digestibility in weaned piglets [16]. Similarly, Htoo and Molares (2012) observed that a 35-day supplementation with 1.2% potassium diformate reduced the feed conversion ratio in weaned piglets [17]. However, Poeikhampha and Bunchasak (2011) reported that supplementation with 0.8% potassium diformate for 42 days had no effect on the growth performance of weaned piglets [18]. Additionally, Taube et al. (2009) and Papenbrock et al. (2005) reported the prophylactic effects of 1.2% potassium diformate in weaned piglets infected with *Salmonella* Derby [19,20] or *Escherichia coli* [19]. Canibe et al. (2001) evaluated the effects of 1.8% potassium diformate administered over 28 days on gastrointestinal pH and microbiota in weaned piglets [21].

However, most studies have focused on the effects of a single dose of potassium diformate on weaned piglets, primarily evaluating growth performance, nutrient digestibility, and antimicrobial activity. In addition, limited information is available regarding antioxidant parameters and digestive enzyme activity in piglets in response to dietary potassium diformate supplementation. Therefore, a 28-day feeding trial was conducted to assess the effects of dietary supplementation with 0, 0.6%, 1.2%, or 1.8% potassium diformate on growth performance, nutrient digestibility, gastrointestinal pH, jejunal morphology, digestive enzyme activity, and antioxidant status in weaned piglets. To the best of our knowledge, the novelty of this study lies in its comprehensive assessment of varying doses of potassium diformate on weaned piglets, along with a correlation analysis of growth performance, nutrient digestibility, gastrointestinal pH, jejunal morphology, digestive enzyme activity, and antioxidant status.

## 2. Materials and Methods

### 2.1. Experimental Design

Twenty-four healthy castrated male weaned piglets (Duroc × Landrace × Yorkshire, 28 days old) were selected and, following a four-day adaptation period, randomly assigned to four treatment groups. Each treatment group comprised six piglets. All piglets were housed in cages (0.8 m height × 1.5 m length × 1.0 m width), each equipped with a heater, a feeder, and a drinker supplying fresh water. Each cage housed a single piglet to allow free movement. A 4-day adaptation period was provided to familiarize the piglets with the experimental environment, including the feeder, basal diet, drinker, and heater. After the 4-day adaptation period, piglets were double-checked for suitability, and the feeding trial commenced. The 4 dietary treatments consisted of a basal diet and 3 diets added with 0.6%, 1.2%, or 1.8% potassium diformate (98% purity, Anhui Zhengzheng Feed Technology Co., Ltd., Bengbu, Anhui, China) in the basal diet. The trial duration was 28 days, during which piglets had ad libitum access to their experimental diets and fresh water. The basal diet (Table 1) is a corn-soybean meal diet, formulated to meet the nutritional requirements for piglets as outlined by NRC (2012) [22]. Potassium diformate was supplemented at 0.6%, 1.2%, or 1.8% to replace zeolite in the premix, which was then incorporated into the complete diets (Table 2). The weight of the piglets was measured at the commencement and conclusion of the experiment. Feed consumption was measured weekly. Growth performance parameters were calculated, and the calculation formulas were referenced from Mesonero Escuredo et al. (2016) [23]: (1) Average daily feed intake = total feed consumption per piglet/experimental days; (2) Average daily gain = (final body weight—initial body weight)/experimental days; (3) Feed conversion ratio = Average daily feed intake/average daily gain.

### 2.2. Sample Collection and Analysis

#### 2.2.1. Nutrient Digestibility

Fresh feces were sampled over the final four days with 10% sulfuric acid (10 mL per 100 g sample). After four days of collection, the feces from individual piglet were thoroughly mixed to form a composite sample. The 4 diets were sampled concurrently with the fecal collection period. The apparent total tract digestibility (ATTD) of dry matter, crude protein, calcium, and phosphous were measured utilizing the acid-insoluble ash method [24]. The contents of dry matter, crude protein, calcium, and phosphorus in feces and diets were analyzed using the method outlined by AOAC (1995) [25]. Finally, the ATTD of the measured nutrients was calculated based on our previous study [26].

#### 2.2.2. Gastrointestinal Digesta pH

Following blood sampling, all piglets were euthanized via sodium pentobarbital administration to obtain digesta samples from the proximal stomach, distal stomach, duodenum, jejunum, ileum, cecum, and colon for pH assessment. Digesta pH was determined using a digital pH meter (AB150 model, Fisher Scientific, Sunnyvale, CA, USA).

#### 2.2.3. Jejunal Morphology

The mid-jejunal segment was excised and fixed in a 4% paraformaldehyde solution for histological evaluation, adhering to the method in our prior investigation [27]. The specimens underwent fixation, dehydration, paraffin embedding, and sectioning. After deparaffinization and rehydration, tissue sections were stained using hematoxylin and eosin (H&E). High-resolution micrographs of the H&E sections were acquired with an microscope (Advanced Microscopy, Bothell, WA, USA). Quantitative analysis of crypt depth and villus height was executed via Image-Pro Plus 6.0 software, with the villus height/crypt depth derived by dividing villus height by crypt depth.

#### 2.2.4. Antioxidant Parameters and Digestive Enzyme Activities in Jejunal Mucosa

Following the collection of jejunal tissue for morphological assessment, an additional jejunal segment was utilized for mucosal sampling. The mucosal layer of the jejunum was carefully scraped with a pre-cooled microscope slide. The jejunal mucosa was homogenized in 0.9% saline solution at a weight-to-volume ratio of 1:9. The homogenate was centrifuged at 3500× *g* and 4 °C for 15 min. And then, the homogenate supernatant was used to determine antioxidant parameters (CAT, T-SOD, T-AOC, and MDA) and digestive enzyme activities (trypsin, α-amylase, and lipase) using commercial kits according to the manufacturer’s instructions (Nanjing Jiancheng, Nanjing, China). Protein concentration in the supernatant was measured using a BCA protein assay kit (Nanjing Jiancheng, Nanjing, China).

### 2.3. Statistical Analysis

The sample size of six met the requirement of the statistical power analysis, based on previous studies involving pigs [28,29,30]. Also, a post hoc power analysis was performed using G*Power software (version 3.1.9.3, Heinrich Heine University, Düsseldorf, Germany) to verify that the sample size (n = 6 per group) was adequate to detect a biologically meaningful effect size with 80% power at α = 0.05. The data were analyzed using SPSS 25.0 software (IBM, Chicago, IL, USA). The Shapiro–Wilk test was employed to evaluate normality, while Levene’s test was utilized to assess the homogeneity of variances. When the assumptions were met, a one-way ANOVA followed by Tukey’s HSD post hoc test was conducted. Otherwise, the Kruskal–Wallis test and DSCF test were applied (cecal digesta pH, jejunal crypt depth, and jejunal trypsin activity). Results are presented as mean ± SEM (n = 6). Pearson correlation analysis was further conducted to assess the relationships among the altered parameters associated with growth performance, nutrient digestibility, gastrointestinal pH, jejunal morphology, antioxidant status, and digestive enzyme activity in piglets. Specifically, only parameters demonstrating statistically significant differences among treatment groups in prior analyses (one-way ANOVA followed by Tukey’s HSD post hoc test or Kruskal–Wallis test followed by DSCF test) were included in the correlation analysis. It should be noted that these parameters (cecal digesta pH, jejunal crypt depth, and jejunal trypsin activity), which did not meet the normality distribution assumption, showed no statistical differences among treatment groups and thus were not included in the Pearson correlation analysis. For the parameters included in the Pearson correlation analysis, all data met the normality distribution assumption. Statistical significance was defined as a *p*-value of less than 0.05.

## 3. Results

### 3.1. Growth Performance and Nutrient Digestibility

**Figure 1:**
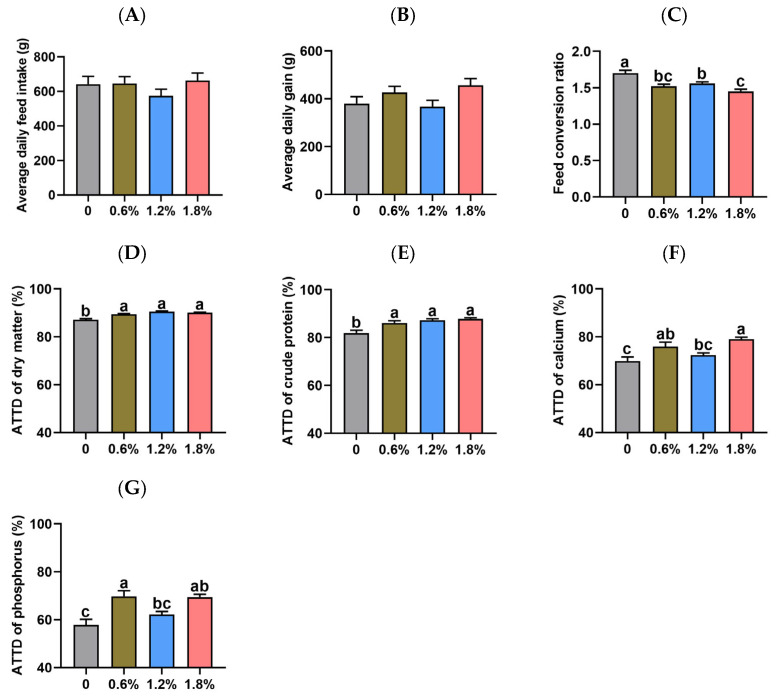
Effect of dietary supplementation with potassium diformate on growth performance and nutrient digestibility in weaned piglets (n = 6, mean ± SEM). (**A**) Average daily feed intake. (**B**) Average daily gain. (**C**) Feed conversion ratio. (**D**–**G**) The apparent total tract digestibility (ATTD) of dry matter, crude protein, calcium, and phosphorus. Different letters on bars indicate significant differences (*p* < 0.05).

The effect of dietary supplementation with potassium diformate on growth performance and nutrient digestibility in weaned piglets is presented in Figure 1. Neither the average daily feed intake nor the average daily gain of weaned piglets was affected by dietary supplementation with 0.6%, 1.2%, or 1.8% potassium diformate (*p* > 0.05). However, the feed conversion ratio of weaned piglets during the 4-week feeding trial was reduced by all three potassium diformate supplementation levels compared to the control group (non-supplemented group) (*p* < 0.05). Specifically, the feed conversion ratio was decreased by 10.59%, 8.24%, and 14.71% in the 0.6%, 1.2%, and 1.8% potassium diformate groups, respectively, relative to the control group. Furthermore, the feed conversion ratio was decreased in piglets fed the 1.8% potassium diformate-supplemented diet compared to those fed the 1.2% potassium diformate-supplemented diet (1.45 ± 0.03 vs. 1.56 ± 0.02; *p* < 0.05). Compared to the control group, piglets fed the three potassium diformate-supplemented diets exhibited significantly higher apparent total tract digestibility (ATTD) of dry matter and crude protein (*p* < 0.05). Relative to the control group, the ATTD of dry matter was increased by 2.69%, 3.98%, and 3.48% in the 0.6%, 1.2%, and 1.8% potassium diformate groups, respectively. Similarly, the ATTD of crude protein was elevated by 5.18%, 6.49%, and 7.24% in the 0.6%, 1.2%, and 1.8% potassium diformate groups, respectively. Additionally, piglets in the 0.6% and 1.8% potassium diformate groups exhibited significantly higher apparent total tract digestibility (ATTD) of calcium (8.58% and 12.96%, respectively) and phosphorus (20.61% and 20.04%, respectively) compared to the control group (*p* < 0.05).

### 3.2. Gastrointestinal Digesta pH

**Figure 2:**
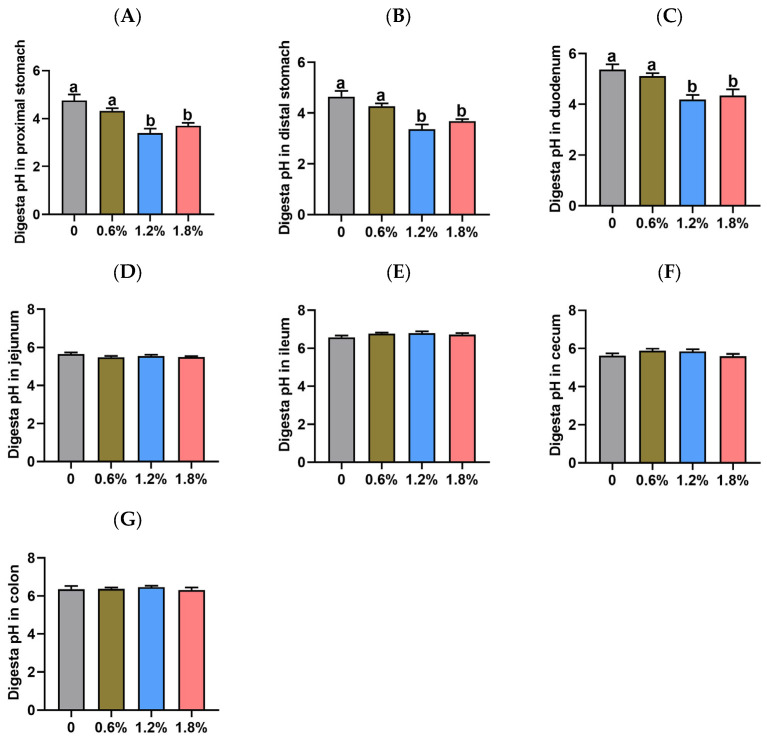
Effect of dietary supplementation with potassium diformate on gastrointestinal digesta pH in weaned piglets (n = 6, mean ± SEM). (**A**–**G**) Digesta pH in the proximal stomach, distal stomach, duodenum, jejunum, ileum, cecum, and colon. Different letters on bars indicate significant differences (*p* < 0.05).

As shown in Figure 2, the digesta pH in the jejunum, ileum, cecum, and colon did not differ among the four treatment groups (*p* > 0.05). Additionally, the digesta pH in the proximal stomach, distal stomach, and duodenum of piglets did not differ between the control group and the 0.6% potassium diformate group (*p* > 0.05). However, dietary supplementation with 1.2% or 1.8% potassium diformate significantly reduced the digesta pH in the proximal stomach, distal stomach, and duodenum of piglets compared to the control group or the 0.6% potassium diformate group (*p* < 0.05). Compared to the control group, dietary supplementation with 1.2% or 1.8% potassium diformate reduced the digesta pH in the proximal stomach (by 28.60% and 22.18%, respectively), distal stomach (by 27.81% and 20.93%, respectively), and duodenum (by 21.97% and 19.09%, respectively) of piglets.

### 3.3. Jejunal Morphology

**Figure 3:**
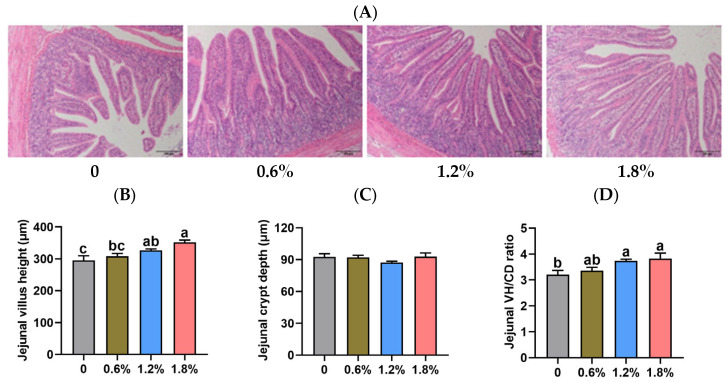
Effect of dietary supplementation with potassium diformate on jejunal morphology in weaned piglets (n = 6, mean ± SEM). (**A**) H&E images. (**B**) Villus height (VH). (**C**) Crypt depth (CD). (**D**) VH/CD ratio. Different letters on bars indicate significant differences (*p* < 0.05).

Figure 3 illustrates the effects of dietary potassium diformate supplementation on jejunal morphology in weaned piglets. The jejunal crypt depth (CD) of piglets was unaffected by dietary treatments (*p* > 0.05). Additionally, supplementation with 0.6% potassium diformate had no significant effect on villus height (VH) or the VH/CD ratio in the jejunum of piglets (*p* > 0.05). However, compared to the control group, dietary supplementation with 1.2% or 1.8% potassium diformate significantly increased both VH and the VH/CD ratio (*p* < 0.05).

### 3.4. Antioxidant Status and Digestive Enzyme Activities in Jejunal Mucosa

**Figure 4:**
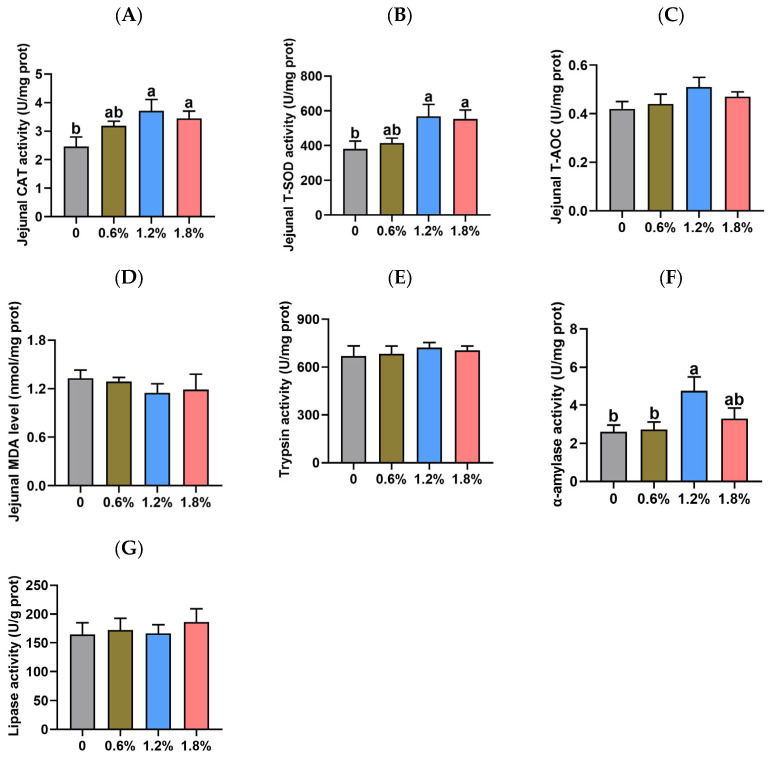
Effect of dietary supplementation with potassium diformate on the antioxidant status and digestive enzyme activities in the jejunal mucosa of weaned piglets (n = 6, mean ± SEM). (**A**) Catalase (CAT) activity. (**B**) Total superoxide dismutase (T-SOD) activity. (**C**) Total antioxidant capacity (T-AOC). (**D**) Malondialdehyde (MDA) level. (**E**) Trypsin activity. (**F**) α-amylase activity. (**G**) Lipase activity. Different letters on bars indicate significant differences (*p* < 0.05).

Figure 4 illustrates the impact of dietary potassium diformate supplementation on the antioxidant status and digestive enzyme activities in the jejunal mucosa of weaned piglets. The T-AOC and MDA level in the jejunal mucosa of piglets remained unaffected by dietary treatments (*p* > 0.05). However, compared to the control group, dietary supplementation with 1.2% or 1.8% potassium diformate significantly increased CAT (by 50.69% and 40.26%, respectively) and T-SOD activities (by 49.35% and 45.47%, respectively) in the jejunal mucosa of piglets (*p* < 0.05). The activities of trypsin and lipase in the jejunal mucosa were not significantly affected by dietary treatments (*p* > 0.05). Compared to the control group or the 0.6% potassium diformate group, supplementation with 1.2% potassium diformate significantly increased α-amylase activity (4.76 ± 0.72 vs. 2.60 ± 0.36 or 2.73 ± 0.39 U/mg prot; *p* < 0.05). However, no significant difference in α-amylase activity was observed between the 1.2% and 1.8% potassium diformate groups (*p* > 0.05).

### 3.5. Correlation Analysis

**Figure 5:**
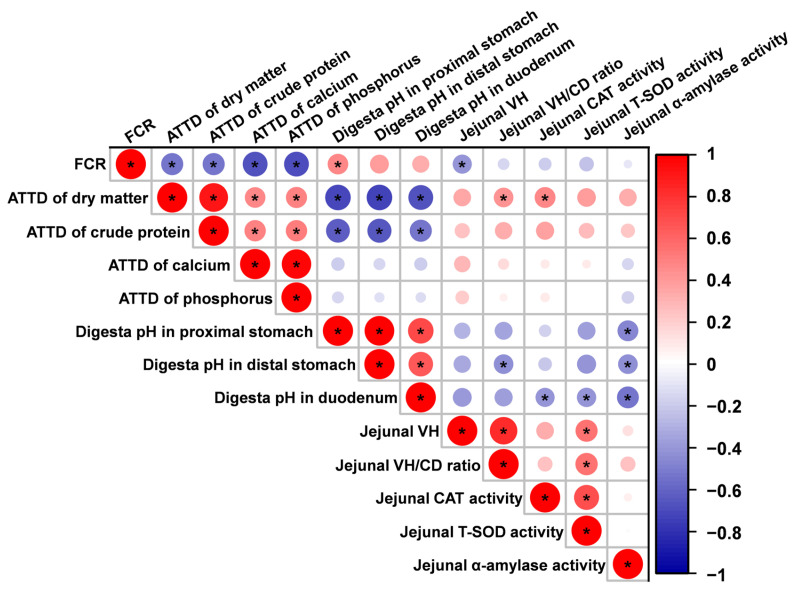
Pearson correlation analysis of altered parameters related to growth performance, nutrient digestibility, gastrointestinal pH, jejunal morphology, antioxidant status, and digestive enzyme activity in piglets. Abbreviations: FCR, feed conversion ratio; ATTD, apparent total tract digestibility; VH, villus height; VH/CD, villus height/crypt depth; CAT, catalase; T-SOD, total superoxide dismutase. * *p* < 0.05.

Figure 5 presents the correlation analysis of altered parameters related to growth performance, nutrient digestibility, gastrointestinal pH, jejunal morphology, antioxidant status, and digestive enzyme activity in piglets. The feed conversion ratio (FCR) exhibited a negative correlation with the apparent total tract digestibility (ATTD) of dry matter, crude protein, calcium, phosphorus, and jejunal villus height (VH), as well as a positive correlation with digesta pH in the proximal stomach (*p* < 0.05). The ATTD of dry matter showed a negative correlation with digesta pH in the proximal stomach, distal stomach, and duodenum, and a positive correlation with the jejunal VH/crypt depth (VH/CD) ratio and jejunal catalase (CAT) activity (*p* < 0.05). The ATTD of crude protein exhibited a negative correlation with digesta pH in the proximal stomach, distal stomach, and duodenum (*p* < 0.05).

## 4. Discussion

The aim of this investigation was to assess the impact of dietary potassium diformate supplementation at dosages of 0, 0.6%, 1.2%, or 1.8% on growth performance, gastrointestinal pH, jejunal morphology, digestive enzyme activity, and antioxidant capacity in weaned piglets. In this study, all three potassium diformate supplementation levels significantly reduced the feed conversion ratio (FCR) of weaned piglets during the 4-week feeding trial relative to the control group. Notably, in the present study, piglets fed the 1.8% potassium diformate-supplemented diet demonstrated a lower FCR compared to those fed the 1.2% supplemented diet. Consistently, the FCR of piglets was reduced by dietary supplementation with 1% potassium diformate for 35 days in the study by Zhou et al. (2015) [16]. In addition, Htoo and Molares (2012) also found that dietary supplementation with 1.2% potassium diformate for 35 days decreased the FCR of piglets [17]. The findings demonstrate that a dosage of 1.2% potassium diformate reduced FCR, whereas a higher dosage of 1.8% further decreased FCR in weaned piglets.

Another potential benefit of potassium diformate is its role in improving nutrient digestibility [31,32]. In our study, the correlation analysis showed that FCR exhibited a negative correlation with the ATTD of dry matter, crude protein, calcium, phosphorus. Piglets receiving diets supplemented with potassium diformate at three different levels displayed significantly greater ATTD of dry matter and crude protein relative to the control group. Furthermore, those administered 0.6% and 1.8% potassium diformate exhibited enhanced ATTD of calcium and phosphorus in comparison to the control group. Reese et al. (2002) also found that dietary supplementation with potassium diformate elevated apparent fecal digestibility of ash and potassium in growing-finishing pigs [33]. The results further substantiate the beneficial additive effects of potassium diformate, reinforcing its role in improving growth performance.

Weaned piglets have not yet fully developed their gastric acid secretion function, leading to limited gastric acid secretion capacity during the early stages [34]. Supplementation with organic acids supports digestive functions by lowering gastrointestinal pH in piglets [35]. In the present study, we supplemented 0.6%, 1.2%, and 1.8% potassium diformate in the diets of weaned piglets and found that dietary supplementation with 1.2% or 1.8% potassium diformate reduced the digesta pH in the proximal stomach, distal stomach, and duodenum of piglets compared to the control group or the 0.6% potassium diformate group. However, the digesta pH in the proximal stomach, distal stomach, and duodenum of piglets did not differ between the control group and the 0.6% potassium diformate group. These findings suggest that supplementation with 0.6% potassium diformate is insufficient to alter the pH of the intestinal contents in piglets, whereas adding 1.2% or 1.8% can reduce the pH value in the anterior part of the gastrointestinal tract. Interestingly, regardless of the dosage of potassium diformate supplementation, the digesta pH in the jejunum, ileum, cecum, and colon did not differ among the four treatment groups. This may be explained by the fact that unprotected organic acids were generally considered effective in lowering the pH in the anterior gastrointestinal tract but were neutralized by bile in the hindgut segment [36].

The jejunum is the primary site of nutrient absorption in pigs [37]. Therefore, we assessed jejunal morphology in piglets. Villus height (VH), crypt depth (CD), and the VH/CD ratio are commonly used indicators of small intestinal morphology [38]. In the current study, dietary supplementation with 1.2% or 1.8% potassium diformate significantly increased both VH and the VH/CD ratio compared to the control group. The improved intestinal morphology may be attributed to the liberation of formic acid and potassium formate in the stomach following potassium diformate ingestion, with a significant portion entering the small intestine within 4 h postprandially [33]. Importantly, potassium formate has been shown to enhance intestinal morphology in piglets [39]. However, 0.6% potassium diformate supplementation had no significant effect on VH and the VH/CD ratio in the jejunum of piglets, which suggested dose-dependent effects of organic acids. These results align with gastrointestinal pH data, suggesting that 0.6% potassium diformate may be insufficient to promote intestinal development.

Digestive enzyme activity and antioxidant capacity serve as important indicators of intestinal health in weaned piglets [40,41] and can also reflect the additive effects of organic acids [42]. As such, we measured the digestive enzyme activity in jejunal mucosa of piglets. Compared to the control group or the 0.6% potassium diformate group, supplementation with 1.2% potassium diformate significantly increased α-amylase activity. The jejunal α-amylase activity exhibited a negative correlation with digesta pH in the proximal stomach, distal stomach, and duodenum. These findings suggest that elevated α-amylase activity may be attributed to the reduced pH environment in the anterior gastrointestinal tract. Guo et al. (2025) reported that dietary supplementation with organic acids increased α-amylase activity in the jejunal digesta of piglets [43], which supports this explanation. Compared to the control group, dietary supplementation with 1.2% or 1.8% potassium diformate significantly increased CAT and T-SOD activity in the jejunal mucosa of piglets. The correlation analysis revealed negative correlations between CAT activity and duodenal digesta pH, as well as between T-SOD activity and duodenal digesta pH. Cai et al. (2024) found that dietary supplementation with organic acids increased CAT activity in the plasam, and *SOD1* mRNA expression in the jejunum of weaned piglets [44]. Similarly, Li et al. (2023) observed that dietary supplementation with compound organic acid, primarily formic acid, elevated serum T-SOD activity in weaned piglets [45]. These findings indicate that potassium diformate supplementation improved jejunal antioxidant and digestive enzyme activities, which are linked to a reduction in pH in the anterior gastrointestinal tract.

Lastly, regarding the observed reduction in FCR following dietary supplementation with potassium diformate, this can be attributed to the decrease in gastrointestinal pH and improved intestinal villi development, thereby enhancing nutrient digestibility. This explanation is supported by the negative correlation between FCR and the ATTD of dry matter, crude protein, calcium, phosphorus, and jejunal VH, as well as the positive correlation with digesta pH in the proximal stomach.

## 5. Conclusions

In conclusion, the inclusion of 1.8% potassium diformate in the diet improved the feed conversion ratio in weaned piglets, correlating with increased nutrient digestibility, lower pH levels in the proximal gastrointestinal tract, and enhanced jejunal structural integrity.

## Figures and Tables

**Table 1:** Ingredient omposition and nutrient content of the basal diet (as-fed basis).

Ingredient	Content (%)	Nutrient	Content
Corn	27.78	Digestible energy, MJ/kg ^2^	14.79
Expanded corn	35.00	Crude protein, % ^3^	17.71
Soybean meal	8.00	Organic matter, % ^3^	83.57
Extruded soybean	10.00	Calcium, % ^3^	0.81
Whey powder	4.00	Total phosphorus, % ^3^	0.62
Fish meal	2.00	Available phosphorus, % ^2^	0.45
Soybean protein concentrate	5.00	SID Lysine, % ^2^	1.54
Soybean oil	3.00	SID Methionine, % ^2^	0.45
Premix ^1^	5.22	SID Threonine, % ^2^	0.97
Total	100.00	SID Tryptophan, % ^2^	0.28

^1^ The premix supplies the following nutrient values per kilogram of diet: vitamin A, 6000 IU; vitamin D_3_, 400 IU; vitamin E, 10 IU; vitamin K_3_, 2 mg; vitamin B_1_, 0.8 mg; vitamin B_6_, 2.4 mg; vitamin B_12_, 0.012 mg; folic acid, 0.2 mg; niacin, 14 mg; pantothenic acid, 10 mg; Zn, 80 mg; Mn, 40 mg; Fe, 100 mg; Cu, 10 mg; I, 0.3 mg; Se, 0.3 mg. ^2^ Calculated values. ^3^ Analyzed values. Abbreviations: SID, standardized ileal digestibility.

**Table 2:** Experimental treatments with different dosages of potassium diformate.

Items	Basal Diet	0.6% Potassium Diformate-Supplemented Diet	1.2% Potassium Diformate-Supplemented Diet	1.8% Potassium Diformate-Supplemented Diet
Potassium diformate level	0	0.6%	1.2%	1.8%
Zeolite level	1.8%	1.2%	0.6%	0
Total ^1^	1.8%	1.8%	1.8%	1.8%

^1^ The 1.8% supplement was prepared using potassium diformate and zeolite. This supplement was then added to the premix to achieve a 5.22% premix. The 5.22% premix was subsequently incorporated into the complete diets.

## Data Availability

Data will be made available upon request.

## Abbreviations

ATTD	Apparent total tract digestibility
CAT	Catalase
CD	Crypt depth
FCR	Feed conversion ratio
H&E	Hematoxylin and eosin
MDA	Malondialdehyde
SID	Standardized ileal digestibility
T-AOC	Total antioxidant capacity
T-SOD	Total superoxide dismutase
VH	Villus height
VH/CD	Villus height/crypt depth
